# Monte Carlo analysis of energy deposition and X‐ray fluence in cylindrical anode systems

**DOI:** 10.1002/acm2.70262

**Published:** 2025-09-30

**Authors:** M. K. M. Alharbi, Abel Zhou, Rob Davidson, Mark Naunton, Chandra Makanjee

**Affiliations:** ^1^ University of Canberra Bruce ACT Australia; ^2^ Taif University Taif Saudi Arabia; ^3^ Singapore Institute of Technology Singapore Singapore; ^4^ College of Medicine, Nursing & Health Sciences Fiji National University Suva Fiji

**Keywords:** cylindrical anode, focal spot distortion, Monte Carlo simulation, X‐ray fluence, X‐ray tube

## Abstract

**Background:**

Cylindrical anode X‐ray systems are increasingly used in multisource imaging; however, electron beam interactions with the curved anodes cause geometric distortions that alter energy deposition and X‐ray emission. Understanding these effects is key to optimizing system performance.

**Purpose:**

This study uses Monte Carlo (MC) simulations to examine how electron beam size, anode radius, and polar angle influence energy deposition and X‐ray fluence in cylindrical anode setups, and to quantify the distortions and energy redistribution for improved X‐ray generation efficiency and beam stability.

**Methods:**

MC simulations were performed with electron beams (0.5 × 0.5 mm and 2 × 2 mm, 120 keV) on cylindrical tungsten anodes with radii from 1 to 5 cm and polar angles from 20.5° to 71.8°. Energy deposition profiles, dimensions, and photon fluence distributions were analyzed using the FLUKA MC package, with mapping in cylindrical coordinates (*r*‐*ϕ*‐*z*).

**Results:**

Energy deposition profiles varied with beam position and anode curvature. The axial full width at half maximum (FWHM) increased by up to 650% at larger polar angles, while the azimuthal FWHM decreased up to 50%. Larger anode radii reduced the azimuthal FWHM by up to 78%, with minimal changes in axial and radial components. Narrower beams (0.5 × 0.5 mm^2^) produced smaller, more symmetric energy deposition profiles on anode surface. Overshoot occurred at small radii and large polar angles, leading to incomplete energy deposition.

**Conclusions:**

Larger anode radii and moderate polar angles minimized energy deposition profiles distortion and improved X‐ray fluence uniformity and production efficiency. Overshooting at small radii and large angles caused deformation, emphasizing the need for precise beam positioning to balance distortion, uniformity, and efficiency. These results define the geometric limits for effective energy deposition profile formation in cylindrical anode systems.

## 1 INTRODUCTION

Multisource X‐ray tube modules using cylindrical anodes have attracted increasing attention because of their potential to enhance imaging performance in medical systems.^1^, ^2^, ^3^, ^4^, ^5^ Compared with conventional stationary anodes, cylindrical anodes offer superior power handling and heat dissipation, making them suitable for high‐performance radiographic applications.^6^ Their geometry enables magnetic beam sweeping, which allows for dynamic focal spot control, as seen in pulsed‐beam sequences^7^ and rapid sequential exposures^8^ for real‐time imaging applications.^2^, ^9^, ^10^, ^11^, ^12^

Although rotating disk anodes have been successfully integrated into conventional X‐ray systems, particularly in CT scanners,^13^, ^14^ their diameter is limited to approximately 200 mm, making them impractical for dense multisource arrays.^15^ Moreover, both stationary and rotating disk anodes lack the geometric adaptability needed for continuous electron beam sweeping across multiple focal positions.

In contrast, cylindrical anodes allow a continuous electron beam to sweep across a curved surface, promoting more uniform heat distribution^16^ and supporting compact, multisource designs. Prior studies have shown that magnetically swept electron beams can dynamically position focal spots across the cylindrical surface.^11^, ^17^, ^18^, ^19^ This feature is particularly beneficial in advanced imaging techniques, such as tomosynthesis,^20^ long‐bone radiography,^21^ and real‐time computed tomography,^16^ where conventional stop‐and‐shoot and slot‐scanner approaches require long acquisition times—up to 12 s for full‐length radiographs^21^, ^22^ compared with 0.05–0.15 s for typical chest X‐rays.^23^, ^24^ A continuously sweeping cylindrical anode could substantially reduce scan times and improve imaging efficiency in clinical environments.

Despite these advantages, research on how the geometry of cylindrical anodes influences focal spot formation and X‐ray fluence characteristics remains limited. Traditional models, including the line‐focus principle, are well‐suited to stationary or angled disk anodes but do not account for the uniform curvature of cylindrical surfaces, which inherently distorts the focal spot.^25^, ^26^, ^27^, ^28^ This distortion can affect beam quality, resolution, and overall cylindrical anode X‐ray system performance. Although Fritz and Livingston^25^ investigated the effect of anode surface curvature on the heel effect, their study did not analyze focal spot deformation, energy profile mapping, or the influence of beam position and geometry on a curved anode surface. Moreover, to the best of our knowledge, no systematic study has examined how variations in electron beam size, anode radius, and polar angle influence focal spot characteristics and energy deposition patterns in cylindrical anode configurations.

To address these gaps in the literature, this study employs Monte Carlo (MC) simulations to evaluate how electron beam size, anode radius, and polar angle affect energy deposition and X‐ray fluence in cylindrical anode systems. The objective is to quantify energy deposition distortions caused by surface curvature and ultimately guide design strategies for stable and efficient X‐ray generation in multisource and swept‐beam imaging applications. MC simulations were selected because they can accurately model electron interactions and spatial energy deposition, allowing systematic analysis across a range of anode geometries and beam configurations under clinically relevant conditions.^29^, ^30^

## 2 METHODOLOGY

### 2.1 Simulation setup

Simulations were performed using FLUKA (v16.10.2024), an MC code designed for modeling particle transport and interactions.^31^, ^32^ MC simulations use random sampling and probabilistic models to track individual particle interactions, making them ideal for studying radiation transport, energy deposition, and X‐ray generation. In this study, FLUKA simulated electron interactions with the anode to precisely model X‐ray production, vary X‐ray target characteristics, and assess beam propagation. Flair (v3.3‐1) was used to create, debug, and execute simulations, with integrated post‐processing tools, such as Gnuplot, for visualizing outputs.^33^ Data were analyzed and plotted using Python and Gnuplot (integrated within Flair). A total of 26 independent simulations were performed (Table 1), with each simulation consisting of 100 cycles and $10^{7}$ electrons per cycle. The setup included an electron source placed 2 cm from the anode. A virtual X‐ray detector (35 cm × 43 cm) was positioned perpendicular to the central ray of the X‐ray beam, with the ray located at the geometric center of the detector and at a source‐to‐detector distance (SDD) of 100 cm.

**TABLE 1:** Simulation parameters for energy deposition mapping on cylindrical tungsten anode.

Simulation group	Beam type	Variable	Range	Fixed parameter	Number of simulations
Central angle variation	CNT‐based	Polar angle (*θ*_s_)	71.8°–20.5° (7 steps)	Anode radius (*r*) = 2 cm	7
	Filament‐based				7
Anode radius variation	CNT‐based	Anode radius (*r*)	1–5 cm (5 steps)	Electron beam distance: *r* + 2 cm energy deposition central angle: *θ*_s_ = 48.6°	5
	Filament‐based				5
Flat anode simulation	CNT‐based	——	2 mm × 2 mm and 0.5 mm × 0.5 mm	Anode tilt = 12°	1
	Filament‐based				1

#### 2.1.1 Electron beam

Electron beams were modeled at 120 keV with a Gaussian spatial distribution in the x‐ and y‐directions, consistent with the emission characteristics of carbon nanotube (CNT) field emitters^34^, ^35^, ^36^, ^37^, ^38^ and thermionic emission from a filament,^39^, ^40^ as detailed in Table 2. CNT‐ and filament‐based electron beams were selected based on their beam sizes (0.5 × 0.5 mm and 2 × 2 mm, respectively^41^, ^42^, ^43^) and their influence on focal spot dimensions. The upper limits of typical focal spot sizes were set to 0.5 mm for CNT‐based emission and 2 mm for thermionic filament emission to examine beam distortion and geometric aberrations and provide an assessment of system performance. The momentum spread under FWHM was estimated from the energy spread $\sigma_{E}$ using the relationship between energy and momentum. For a Gaussian distribution, $\Delta p\left(FWHM\right)=2.355\cdot \sigma_{p}$​, where $\sigma_{p}$​ represents the standard deviation of the momentum spread. The momentum spread relates to the energy spread by $\sigma_{p}$ = $\sigma_{E}/c$, where c represents the speed of light. Therefore, the momentum spread was calculated as $\Delta p\;\left(FWHM\right)=2.355\cdot \sigma_{E}/c$. The azimuthal angular divergence of the beam followed a Gaussian distribution expressed as:

(1) $$P\left(\phi \right)=\frac{1}{\sigma_{\phi }\sqrt{2\pi }}\;e^{-\frac{\phi^{2}}{2\sigma_{\phi }^{2}}},$$

where P(ϕ) and $\sigma_{\phi }$ represent the probability density function for the azimuthal angle *ϕ* and the standard deviation of the angular spread, respectively.^44^ The FWHM of the distribution is related to $\sigma_{\phi }$ by $\Delta \phi \;\left(FWHM\right)=2.355\cdot \sigma_{\phi }$. A collimated beam with an angular divergence of Δ*ϕ* (FWHM) = 0.005 mrad was employed to minimize angular spread, ensure consistency, and reduce variance in the simulation.

**TABLE 2:** Characteristics of simulated electron beam.

	Filament‐based beam	CNT‐based beam
Electron source mechanism	Thermionic emission (heat‐driven)	Field emission (electric field‐driven)
Energy (keV)	120 keV	120 keV
Number of electrons (N)	1 × 10^9^	1 × 10^9^
Beam source size (mm)	2 mm × 2 mm	0.5 mm × 0.5 mm
Beam divergence Δ*ϕ* (FWHM)	0.01 mrad × 0.01 mrad	0.005 mrad × 0.005 mrad
Momentum spread Δp (FWHM)	$5\times 10^{-6}\;GeV/c$	$5\times 10^{-8}\;GeV/c$
Simulation conditions	Gaussian distribution	Gaussian distribution

#### 2.1.2 Cylindrical target and electron beam position

MC simulations were conducted to calculate the energy deposition dimensions on a cylindrical tungsten anode with a fixed height (*H* = 5 cm). The beam remained orthogonal to the anode surface throughout all simulations, with a CNT‐based electron beam being used. In the first set of simulations, the variation in the polar angle (*θ*) was investigated using the CNT‐based electron beam. In the second set, the same variation was investigated under identical conditions, but with a filament‐based electron beam. The third set involved varying the anode radius (*r*) between 1 and 5 cm, with the electron beam positioned at a fixed distance of *r* + 2 cm from the anode surface. Energy deposition was located at *θ* = 48,6°, with a CNT‐based electron beam used in this case. The fourth set also involved varying the anode radius under the same conditions, but with a filament‐based electron beam used instead. Additional simulations were conducted on a flat anode using both CNT‐ and filament‐based electron beams (beam dimensions of 0.5 × 0.5 mm and 2 × 2 mm, respectively). For these simulations, the flat anode was tilted at an angle of 12°. The energy deposition dimensions in all simulations were characterized by their FWHM along the *z*‐axis (height), radial direction (*r*), and azimuthal direction (Φ). The simulation conditions are summarized in Table 2.

#### 2.1.3 Scoring and biasing

Photon fluence was scored using the USRTRACK command, configured to record photons in the “VOID” and “detector” regions over an energy range of 20–120 keV. The USRBDX command was employed to calculate angular photon fluence between the same regions using the parameters angular fluence (Φ_2_), linear energy (linE), and linear solid angle (linΩ), with 300 bins for each parameter. Energy deposition was evaluated using the USRBIN command in cylindrical coordinates (*r*‐*ϕ*‐*z*) to map energy deposition on the curved anode surface. Photon distributions in three‐dimensional space were scored in Cartesian coordinates (*x‐y‐z*) to capture photon propagation within the setup, including the region surrounding the source and extending toward the detector. All scoring used bins with a resolution of 300 × 300 × 300 for high spatial and energy resolution. A biasing technique^45^, ^46^ was applied using the “BIASING” command with a splitting factor of 5. For particles crossing from region *i* to region *j*, the following rules were applied.

**FIGURE 1:**
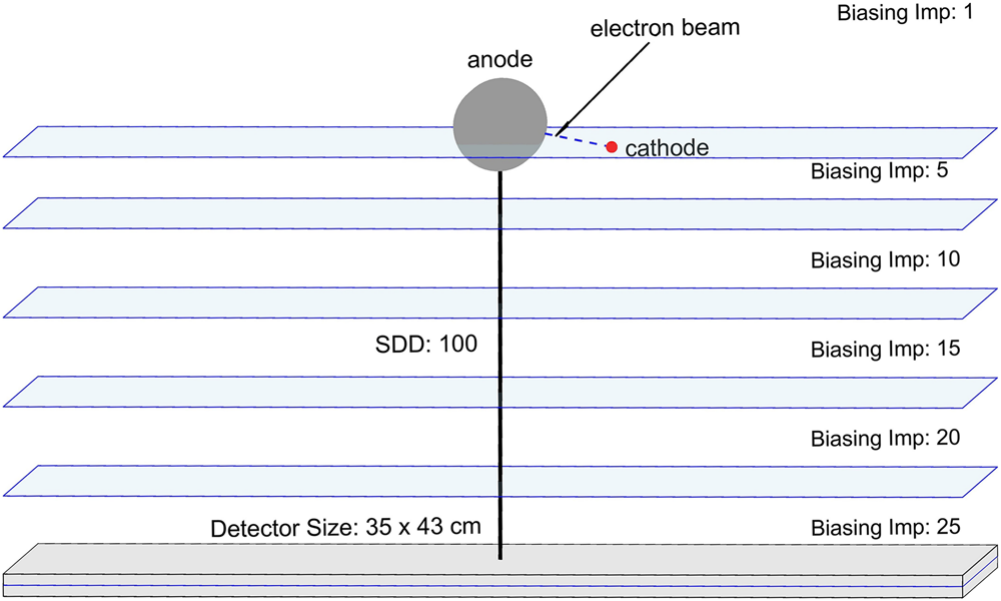
Simplified three‐dimensional sketch of the cylindrical tungsten anode, cathode, and detector, highlighting biasing regions with annotated importance values (Imp).

For $I_{j}>\;I_{i}$ (splitting),

(2) $$n_{s}=\frac{I_{j}}{I_{i}},$$

where $n_{s}$ denotes the splitting factor, representing the number of particle tracks generated in the new region.

(3) $$w_{j}=\frac{w_{i}}{n_{s}},$$

where $w_{j}$ and $w_{i}$ represent the weight assigned to each split track and the original weight of the particle before splitting, respectively (Figure 1).

#### 2.1.4 Uncertainty in the simulation and validation

MC simulations were conducted using FLUKA, a widely established and validated radiation transport code.^31^, ^32^ Since this study involves the design of a novel X‐ray generator with no experimental validation data, the simulations were executed with 100 independent cycles—10 times higher than the standard recommendation for statistical uncertainty estimation.

FLUKA estimates the variance of the mean of a quantity *x* (e.g., fluence) across 𝑁 batches using the following formula:

(4) $$\sigma_{\langle x\rangle }^{2}=\frac{1}{N-1}\;\left[\frac{\sum_{1}^{N}n_{i}x_{i}^{2}}{n}-{\left(\frac{\sum_{1}^{N}n_{i}x_{i}}{n}\right)}^{2}\right]$$

where n_i_ is the number of histories in the *i* ^th^ batch, *n* = ∑*n_i_* is the total number of histories, $x_{i}=\left(\sum_{j=1}^{n_{i}}\frac{x_{ij}}{n_{i}}\right)$ is the average value of x in the *i* ^th^ batch, and *x_ij_* is the contribution to *x* from the *j* ^th^ history in the *i* ^th^ batch. This expression calculates the mean of the squares minus the square of the mean, normalized by (N−1). Statistical uncertainty was estimated by propagating voxel‐wise percentage errors reported in the FLUKA output across all non‐zero voxels. For further validation, a typical fixed anode X‐ray tube with a 12° target tilt, 120 kVp, and an Al X‐ray filter with a thickness of 4.13 mm was simulated and compared to three different datasets with the same target tilt and filter thickness, as detailed in Table 3. The comparison focused on the produced X‐ray spectra, including their shape and energy peaks. To quantify agreement between FLUKA and reference spectra, the root‐mean‐square error (RMSE) was calculated between each normalized spectrum and the FLUKA output. RMSE was computed over all energy bins and reflects the overall deviation in spectral shape.

**TABLE 3:** Datasets used for comparison.

Data source	Data type
SpekPy^47^	SpekPy dataset comprises computer generated data from SpekPy v2, which is a free, open‐source Python toolkit that accurately models on‐ and off‐axis X‐ray tube spectra for tungsten and molybdenum targets.
AAPM TG‐195 data^48^	The AAPM TG‐195 dataset consists of multiple simulations outputs for dose, energy fluence, scatter, and spectra computed using EGSnrc, GEANT4, MCNPX, and PENELOPE under standardized conditions.
TASMIP^49^	TASMIP is a polynomial‐based spectral model that generates 1 keV‐resolution tungsten anode X‐ray spectra from 30 to 140 kV by interpolating modified experimental Fewell data without physical assumptions.

#### 2.1.5 X‐ray production efficiency calculation

The X‐ray production efficiency was evaluated for each X‐ray tube configuration by calculating the fraction of incident electron energy converted into useful photon energy reaching a 43 × 35 cm^2^ detector placed 100 cm from the source. Only photons with energies ≥ 33 keV were included in the analysis. The efficiency was computed using the following formula:

(5) $$\eta =\frac{\Phi \cdot A\cdot {\bar{E}}_{\gamma }}{E_{0}}\;$$

where *Φ* is the fluence, A is the detector area (1505 cm^2^), ${\bar{E}}_{\gamma }$​ is the average photon energy (keV), and $E_{0}$​ is the incident electron energy (120 keV).

#### 2.1.6 Data collection, analysis, and computational setup

Simulation runs were performed across defined parameter sets to evaluate energy deposition characteristics and X‐ray emission behavior. The angular distribution of X‐rays was extracted using the USRBDX command, followed by detailed energy deposition mapping. The simulations were executed on an Ubuntu 24.04 system (11th Gen Intel Core i9‐11900H processor with 8 cores and 16 threads, base clock speed = 2.50 GHz, and boost clock speed = 4.90 GHz).

#### 2.1.7 Energy deposition and X‐ray emission analysis

**FIGURE 2:**
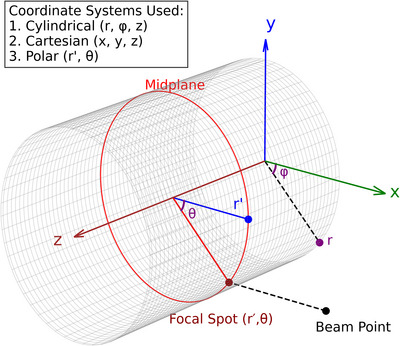
Coordinate systems and geometry used for defining the focal spot on a cylindrical anode.

Energy deposition positions were described using *θ*, which defines the angular location of the interaction point along the cylindrical curvature, and the azimuthal angle (*ϕ*), which determines the angular range of interaction within the cylindrical coordinate system (Figure 2). For a horizontally oriented cylinder, the interaction region was confined to a quarter of the cylindrical surface located on the rear‐left side, within a specific angular sector along the azimuthal direction. In the simulations, *θ* was systematically varied to shift the focal spot location along the curvature while maintaining it within this azimuthal range. Additionally, the anode radius and electron beam size were adjusted to investigate their effect on the geometric distortion of the focal spot and the corresponding X‐ray emission characteristics. The X‐ray fluence distribution was scored at the detector to assess variations in intensity and coverage arising from positional and geometric changes.

## 3 RESULTS AND DISCUSSION

### 3.1 Validation of simulations

**FIGURE 3:**
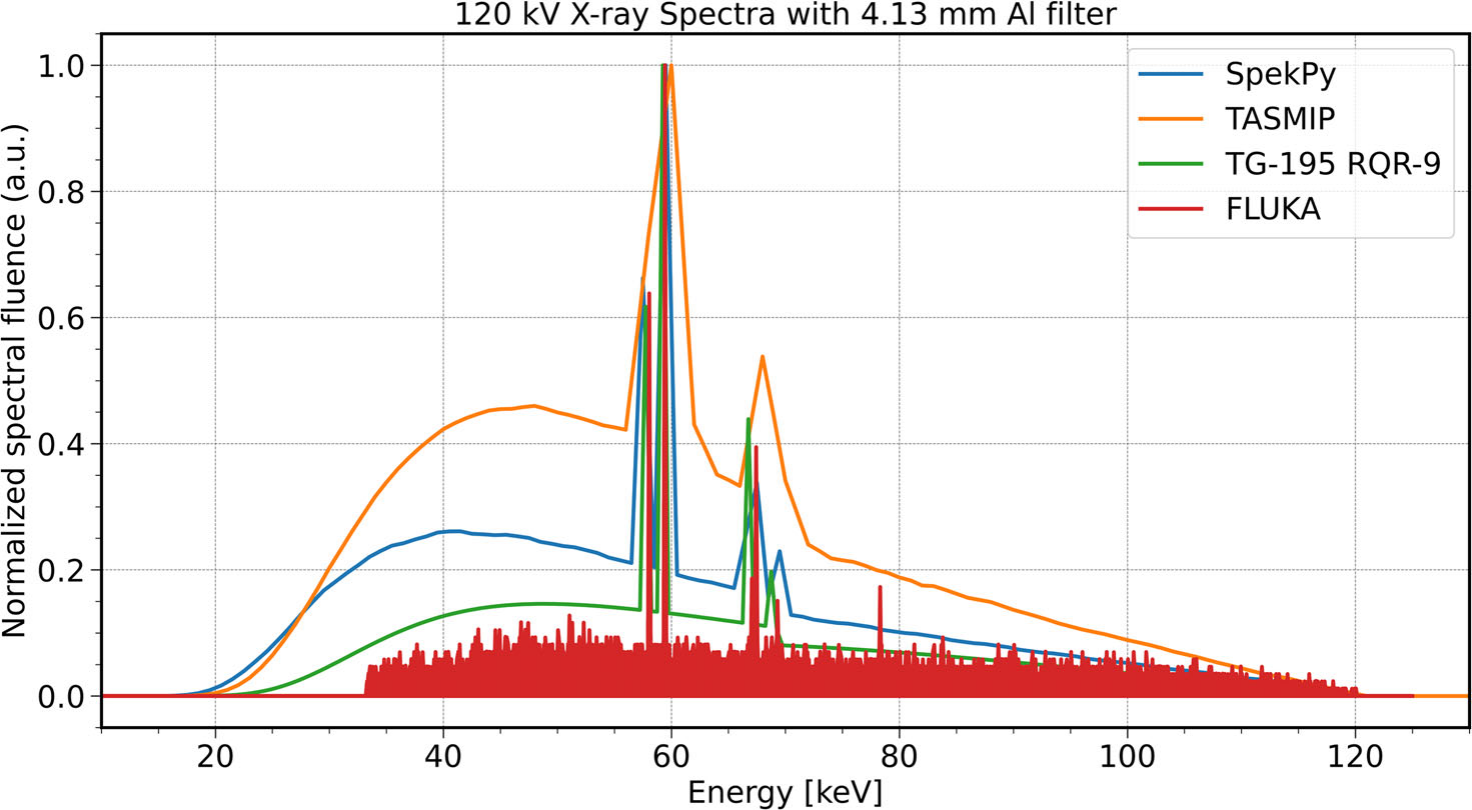
Comparison of the FLUKA‐simulated 120 kV X‐ray spectrum with SpekPy, TASMIP, and TG‐195, showing agreement in peak position and differences in spectral width and beam hardness.

FLUKA was validated against SpekPy, TASMIP, and TG‐195 using spectral metrics and visual comparison. The peak energy was consistent across all models (FLUKA: 59.44 keV; others: 59.25–60.00 keV), confirming correct characteristic line placement. FLUKA showed the narrowest FWHM (0.18 keV) compared to TG‐195 (2.12 keV), SpekPy (3.36 keV), and TASMIP (12.49 keV), indicating better line resolution. The mean energy was highest in FLUKA (66.83 keV), reflecting a harder beam compared to TG‐195 (61.18 keV), TASMIP (59.13 keV), and SpekPy (57.45 keV). FLUKA also had the highest > 40 keV photon fraction (94.41%) and total spectral area (59.6), indicating greater beam filtration and fluence. Figure 3 confirms this, showing FLUKA with a sharp peak and minimal low‐energy tail, while the other models appear broader and softer. RMSE values (TG‐195: 0.103, SpekPy: 0.167, TASMIP: 0.281) show the closest agreement between FLUKA and TG‐195. These results validate FLUKA's accuracy in reproducing a filtered 120 kV clinical X‐ray spectrum. Moreover, the average simulation errors were ∼0.56% for the CNT and ∼0.61% for the filament source in X‐ray fluence, with energy deposition uncertainties below ∼0.004% for both. Complete data are provided in the supplementary materials Table S1.

### 3.2 Energy deposition at focal spot area

**FIGURE 4:**
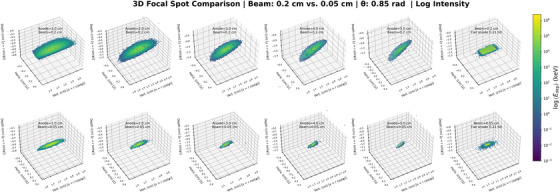
Logarithmic energy maps for radii ranging from 1.0 cm to 5.0 cm under beam diameters of 0.2 cm (top) and 0.05 cm (bottom). A flat anode tilted at 12° is included for comparison.

The deposition profile shape is strongly influenced by anode radius, beam diameter, and incidence angle. At small radii (1.0–2.0 cm), energy deposition is elongated and asymmetric, particularly for the 2‐mm beam. Increasing the radius to 5 cm reduces this distortion, making the spot more compact and symmetric (Figure 4). The 0.5‐mm beam produces consistently localized deposition across all radii. A flat anode tilted at $12^{\circ }$ is included for comparison, showing minimal distortion and demonstrating the effect of cylindrical geometry on energy deposition profile deformation.

**FIGURE 5:**
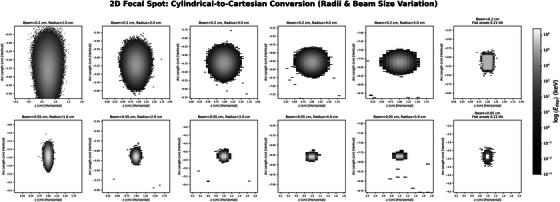
Logarithmic energy maps for a 2.0 cm radius anode with beam angles ranging from 20.5° to 71.8°. Beam diameters: 0.2 cm (top) and 0.05 cm (bottom). A flat anode with a 12° tilt is included for comparison.

With the radius fixed at 2 cm, an increase in beam position (polar angle) from 20.5° to 71.8° results in progressive elongation of the energy deposition profiles along the arc length and increased asymmetry owing to surface curvature (Figure 5). This effect is more pronounced for the 0.2‐cm beam, which exhibits trailing energy deposition at higher angles. At lower angles (≤ 26.7°), the energy deposition becomes more confined and symmetric. The 0.5‐mm beam maintains compact energy deposition profiles across all angles, showing reduced sensitivity to angular distortion. The flat anode with a 12° tilt serves as a reference case, clearly illustrating the distortion introduced by cylindrical shaping.

Energy deposition distortion is minimized in the case of narrow beams (≤ 0.05 cm), low polar angles (≤ 26.7°), and large anode radii (≥ 5.0 cm). In the case of the flat anode, it is confirmed that the observed deformation is a geometric consequence of the finite anode radius.

### 3.3 Arc length and geometrical overshoot

**FIGURE 6:**
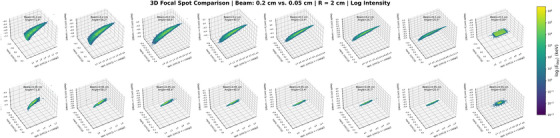
Deposition area distributions for varying anode radii. The arc length domain decreases with radius, and the shape evolves from elongated too isotropic. Overshoot is most apparent at smaller radii.

The reconstructed energy deposition distributions, derived from cylindrical‐to‐Cartesian coordinate transformations, exhibit systematic spatial variation along the arc length direction. In this transformation, the azimuthal coordinate Φ is converted into a linear spatial dimension—arc length—using the relation:

(4) arclength=r·ϕ,

where *r* is the anode radius. This mapping enables a planar representation of cylindrical geometries, allowing for quantitative evaluation of the energy deposition profile shape and dimensional variation. As the anode radius increases, the angular extent of energy deposition decreases, resulting in a reduced arc length domain and modified deposition geometry (Figure 6). At small radii (e.g., 1.0 cm), energy deposition elongates along the arc length axis and shows pronounced asymmetry. As the radius increases to 3.0–4.0 cm, the distribution becomes more isotropic, with a reduced aspect ratio. At 5.0 cm, the energy deposition becomes laterally compressed, and no further improvement in symmetry is observed.

**FIGURE 7:**
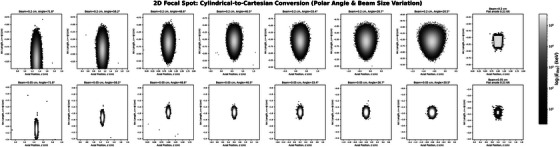
Deposition area distributions for varying polar angles. The arc length domain increases with angle, with elongation, distortion, and overshoot artifacts more pronounced at higher angles.

Variation in polar angle *θ* at a fixed anode radius and beam size also influences the arc length domain, but with distinct effects on energy deposition geometry (Figure 7). As Φ increases, the arc length domain expands proportionally, resulting in elongation of the focal region along the arc length axis. At lower angles (e.g., 20.5°), the distribution is spatially limited. At higher angles (e.g., 71.8°), the distribution becomes highly anisotropic, with substantial elongation and increased geometric distortion.

Overshoot is identified as discontinuities at the arc length boundaries and is most apparent at small anode radii and large polar angles. This effect is attributed to geometric constraints that prevent complete electron interception by the anode surface. Overshoot is most prominent under conditions of high anode curvature (i.e., small radius) or large beam incidence angles (i.e., steep trajectories) and decreases with increasing anode radius.

### 3.4 Effect of anode radius (*r*) on energy deposition profile geometry

**FIGURE 8:**
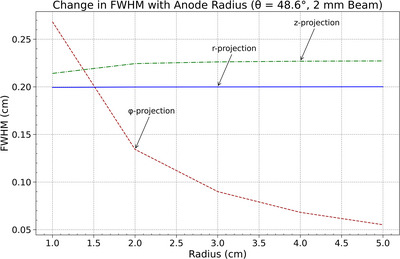
FWHM variation with anode radius (*r*) for azimuthal (*ϕ*), radial (*R*), and axial (*z*) projections, illustrating the decrease in azimuthal spread and the stability of radial and axial spreads for a 2 mm beam.

**FIGURE 9:**
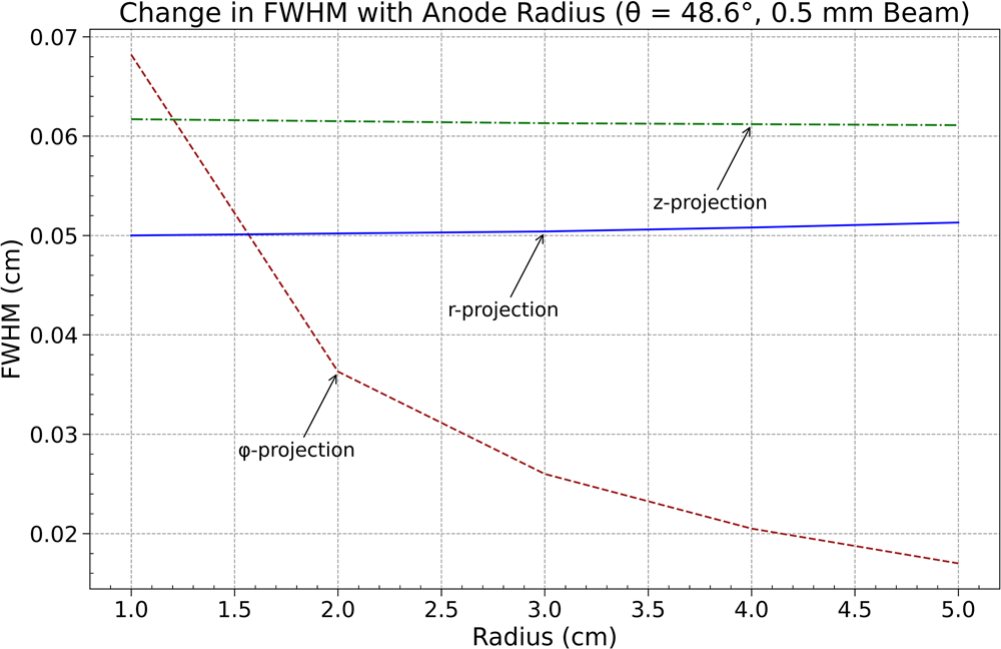
FWHM variation with anode radius (*r*) for azimuthal (*ϕ*), radial (*R*) and axial (*z*) projections, illustrating the decrease in azimuthal spread and the stability of radial and axial spreads for a 0.5 mm beam.

The dependence of focal spot dimensions on anode radius was evaluated for beam widths of 2 mm and 0.5 mm using FWHM in the radial (𝑟), azimuthal (*ϕ*), and axial (*𝑧*) projections at a fixed polar angle *θ* = 48.6°. As shown in Figures 8 and 9, the azimuthal FWHM decreased with increasing anode radius, indicating improved spatial resolution along the *ϕ*‐direction. The axial and radial FWHM values remained approximately constant across the radius range for both beam widths, indicating no significant variation in spatial resolution along the *z*‐ and *r*‐directions.

The observed trend in the azimuthal projection aligns with geometric effects introduced by the changing surface curvature. As the anode radius increases, the local surface at the beam incidence point flattens, reducing projection spread in the azimuthal direction. The 0.5‐mm beam consistently produced lower FWHM values but followed the same directional trends. The relative stability of the radial and axial projections suggests these components are less sensitive to surface curvature within the investigated range.

### 3.5 Energy deposition FWHM versus polar angle

**FIGURE 10:**
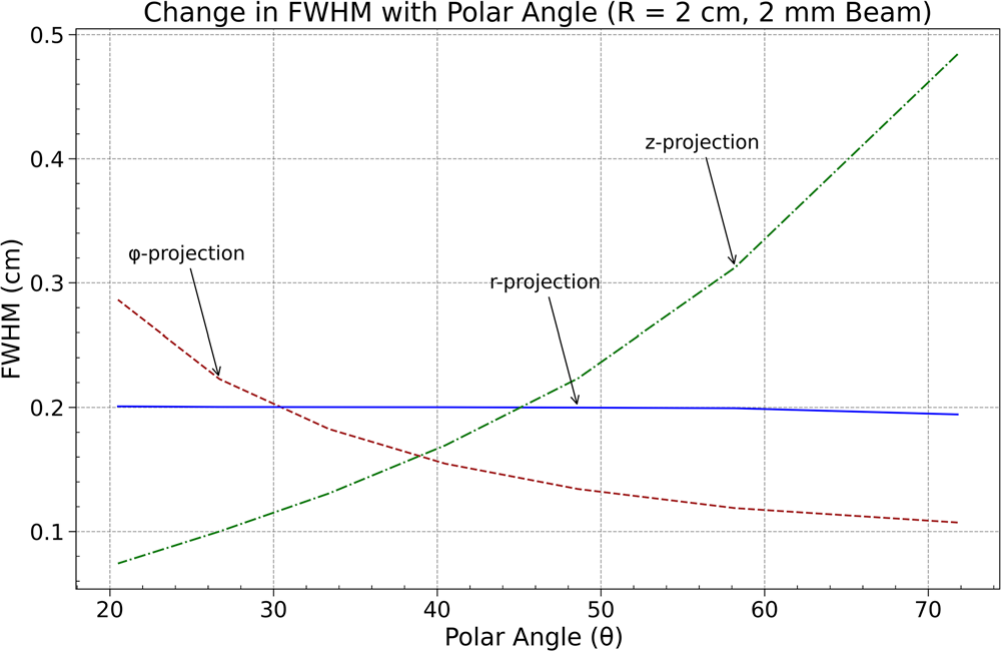
Change in FWHM with polar angle (*θ*) for 2 mm beam (*r* = 2 cm): axial FWHM increases, azimuthal FWHM decreases, and radial FWHM remains constant.

**FIGURE 11:**
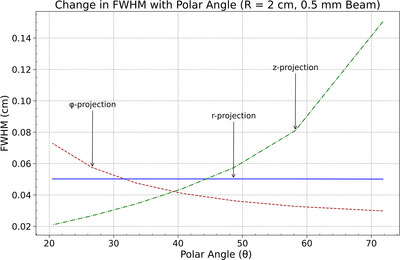
Change in FWHM with polar angle (*θ*) for 0.5 mm beam (*r* = 2 cm): axial FWHM increases, azimuthal FWHM decreases, and radial FWHM remains constant.

Energy deposition dimensions as a function of beam position along the cylindrical anode surface were evaluated for beam widths of 2 mm and 0.5 mm using FWHM in the radial (*r*), azimuthal (*ϕ*), and axial (𝑧) projections as a function of polar angle *θ*. In both cases, the axial FWHM increased with *θ*, indicating reduced spatial resolution of the energy deposition along the *z*‐axis at higher polar angles, as shown in Figures 10 and 11. The azimuthal FWHM decreased monotonically with *θ*, suggesting improved spatial resolution along the *ϕ*‐direction. The radial FWHM remained relatively constant across all values of θ for both beam widths, indicating no measurable variation in spatial resolution along the *r*‐direction.

These results demonstrate consistent anisotropy in energy deposition resolution across beam widths, driven by projection geometry as the beam position varies along the curved anode. The narrower beam produced smaller FWHM values overall but preserved the same directional trends. The intersection of the axial and azimuthal FWHM curves shifted with beam size, marking the location where approximately isotropic resolution was achieved. The total energy depostion at the focal spot area is provided in Supplementray Materials Figure S2.

### 3.6 Energy deposition at 100 SDD

Energy deposition at a SDD of 100 cm was analyzed in relation to polar angle and anode radius. The energy deposited was integrated over the *x*–*z* plane at each axial (*y*) coordinate, resulting in one‐dimensional profiles along the *y*‐axis. This projection isolates the energy distribution along the *y*‐direction in the detector plane, allowing for a quantitative evaluation of the anode heel effect. The analysis highlights how geometric parameters—specifically polar emission angle and anode curvature—affect the magnitude and uniformity of energy deposition along the axial direction.

#### 3.6.1 Energy deposition: polar angle dependence

**FIGURE 12:**
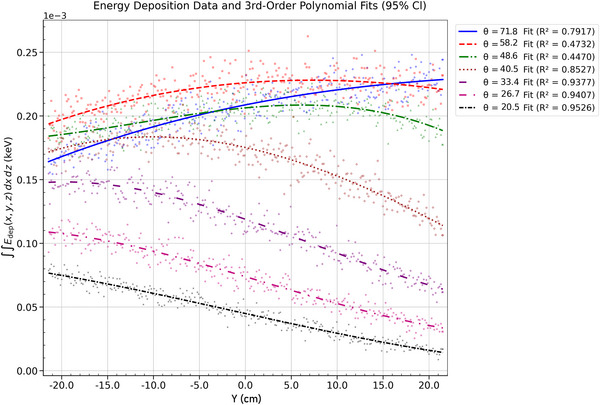
Energy deposition profile for a 2 mm beam (*r* = 2 cm): polar angle dependence with third‐order polynomial fits across *θ*.

**FIGURE 13:**
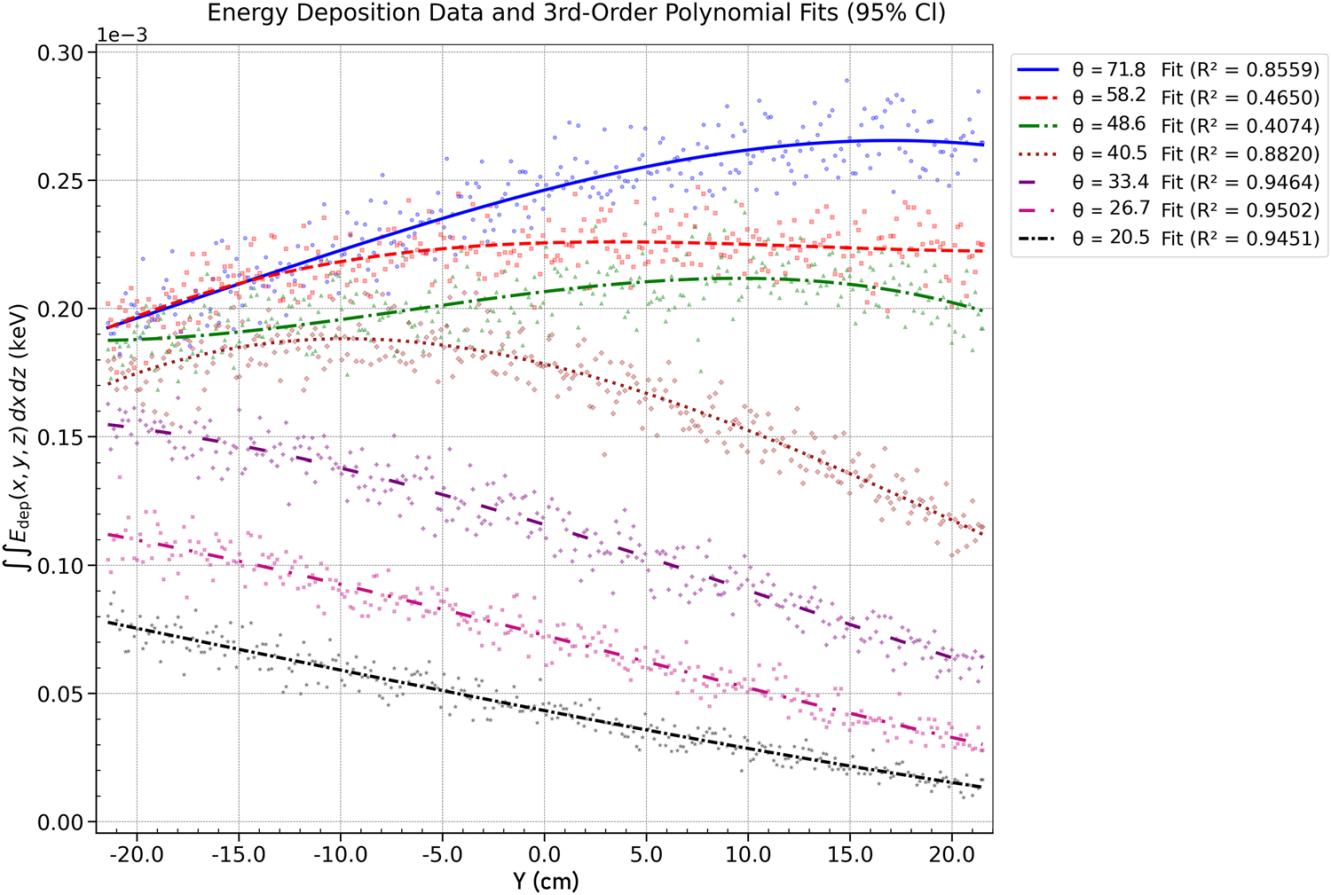
Energy deposition profile for a 0.5 mm beam (*r* = 2 cm): polar angle dependence with third‐order polynomial fits across *θ*.

For both beam sizes, energy deposition is evenly distributed along the *y*‐axis direction at *θ* = 48.6° and 58.2°. At these angles, the geometry of the energy deposition profile and the emission trajectory promote a more uniform energy distribution at the detector. The downward emission trajectory directs the beam toward the detector, increasing fluence. The elongation of the energy deposition profiles—representing the focal spot shape on the *z*–*ϕ* plane of the cylindrical anode—distributes the electron energy across the anode, producing a broader beam. This results in a more uniform X‐ray fluence at the detector, thus reducing the anode heel effect. At lower polar angles (*θ* ≤ 40.5°), energy deposition shifts toward the cathode side (*y* < 0), driven by the X‐ray emission trajectory and interaction pattern within the anode. Intensity decreases at these angles due to reduced energy transfer efficiency as the beam interacts with the anode at shallower angles. Additionally, the 0.5‐mm beam retains slightly higher intensity at *θ* > 48.6°, demonstrating better energy retention than the 2‐mm beam due to reduced geometric overshoot. At *θ* ≤ 48.6° rad, the intensities are comparable for both beam sizes, as illustrated in Figures 12 and 13.

#### 3.6.2 Energy deposition: radius dependence

**FIGURE 14:**
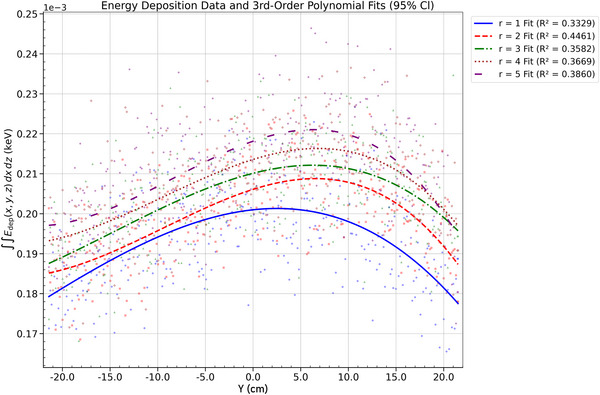
Energy deposition profile for a 2 mm beam (*θ* = 48.6°): radius dependence with third‐order polynomial fits across radii.

**FIGURE 15:**
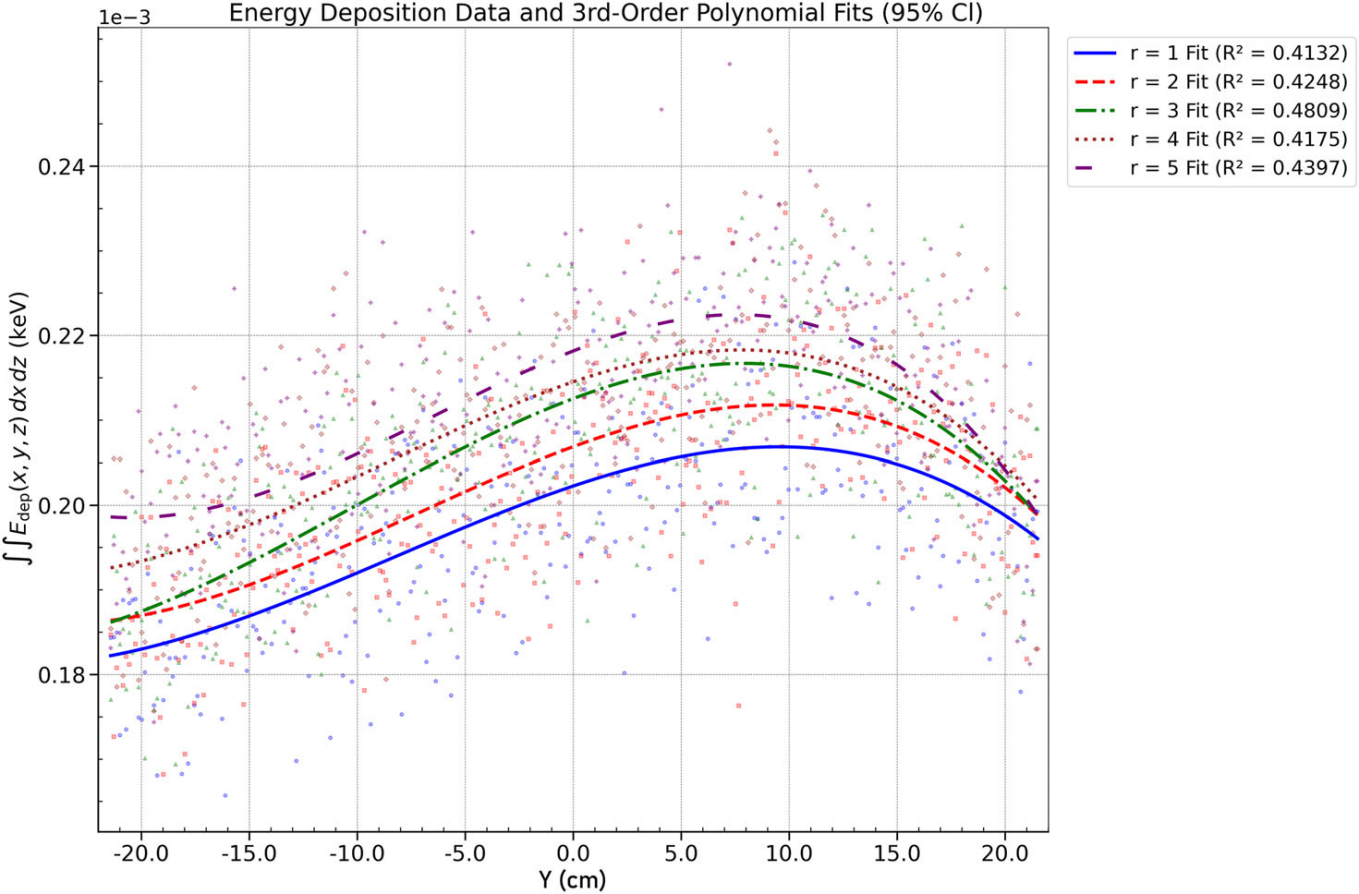
Energy deposition profile for (0.5 mm beam, *θ* = 48.6°): radius dependence with third‐order polynomial fits across radii.

For both beam sizes, increasing *r* significantly amplifies the *y*‐axis energy deposition profiles, indicating greater energy deposition within the anode as curvature decreases, which in turn narrows the angular spread of the X‐ray beam. This effect is likely due to the elongation of energy deposition along the *z*–*ϕ* plane with increasing *r*, as well as potential changes in X‐ray generation efficiency at larger radii. While the energy deposition area on the anode slightly increases with larger radii, the main effect is the redistribution of energy along the *y*‐direction of the detector, resulting from the flattening of the energy deposition geometry. At large radii (*r* = 5 cm), this redistribution causes a slight decrease in deposition uniformity, as energy is spread over a broader range with less consistent intensity. The energy profiles show sharper peaks, reflecting localized deposition variations. These effects are consistent across both beam sizes, with the 0.5‐mm beam maintaining slightly higher peak intensities compared to the 2‐mm beam. This is attributed to the 0.5‐mm beam's narrower focus, which results in more concentrated energy deposition within the anode (Figures 14 and 15).

### 3.7 X‐ray production efficiency

**FIGURE 16:**
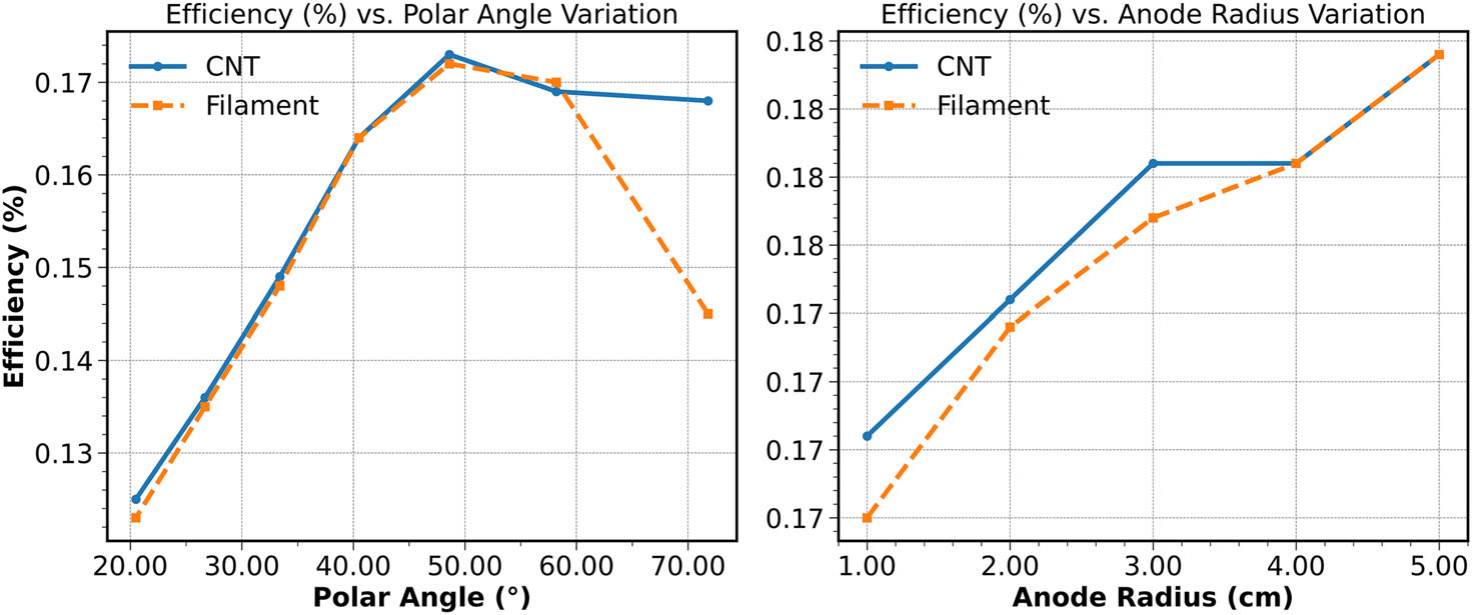
X‐ray production efficiency as a function of polar angle and anode radius for CNT and filament sources.

Efficiency increases with polar angle, from ∼0.125 at 20.5° to a peak of 0.173 at 48.6° for the CNT source and 0.172 for the filament source. Beyond this point, efficiency decreases slightly, dropping to 0.168 for CNT and 0.145 for the filament source at 71.8°. As the anode radius increases, efficiency rises from 0.168 (CNT) and 0.165 (filament) at 1.0 cm to 0.182 for both at 5.0 cm. The flat anode condition results in the lowest efficiency values: 0.115 for CNT and 0.116 for filament. Figure 16 reflects these trends, showing similar behavior for both sources, with the filament source showing a more significant drop at high polar angles. Conperhensive efficiency data are persented in Supplementary Materials Table S1, and the total fluance at detector plane is illustrated in Figure S3.

## 4 CONCLUSIONS

This study used MC simulations to investigate the impact of electron beam size, anode radius, and beam position on energy deposition geometry, energy deposition, and X‐ray fluence in cylindrical anode systems. Both CNT‐based (0.5 mm) and filament‐based (2 mm) beams were modeled across various polar angles and anode radii. Energy deposition profiles, energy deposition, and photon fluence were quantified to assess the geometric distortion caused by surface curvature.

The energy deposition profile showed strong spatial dependence. At a fixed anode radius, increasing the polar angle resulted in a 650% increase in axial FWHM and a 50% reduction in azimuthal FWHM. Increasing the anode radius from 1 to 5 cm reduced azimuthal FWHM by up to 78%, with minimal changes observed in the axial and radial directions. Energy deposition became more uniform at larger radii and higher angles. The 0.5‐mm beam produced smaller, more symmetric energy deposition profiles compared to the 2‐mm beam. Arc‐length mapping showed that energy deposition deformation increased with both polar angle and surface curvature, particularly for wider beams. Overshoot effects were observed at small radii (≤ 2 cm) and large polar angles (*θ* > 58.2°), where electrons exceeded the anode boundary, leading to spatial discontinuities and reduced emission efficiency. These findings suggest that additional modeling is needed to fully characterize boundary interactions. X‐ray fluence analysis confirmed that narrower beams and larger anode radii improve energy confinement and reduce fluence asymmetry. Furthermore, X‐ray production efficiency increased with anode radius, from ∼0.165 to ∼0.182 for both beam types, peaking near 48.6° polar angle before slightly decreasing at higher angles. The lowest efficiencies (∼0.115–0.116) were observed in the flat anode configuration, confirming the role of curvature in enhancing energy‐to‐fluence conversion. These findings collectively support stable focal spot formation, more uniform dose delivery, and improved system performance.

This study provides a framework for direction‐resolved modeling of energy deposition and energy behavior in cylindrical anodes for radiological applications. The results support design choices for multisource, swept‐beam, and real‐time imaging systems, demonstrating that narrow beams, moderate angles, and large radii reduce distortion and improve performance. Future work should evaluate heat dissipation and define operational limits for the source.

## AUTHOR CONTRIBUTIONS

M.K.M. Alharbi: Conceived the research plan, executed the methodology, conducted Monte Carlo simulations, analyzed the data, wrote the manuscript, and performed project administration. Abel Zhou: Co‐conducted Monte Carlo simulations and interpreted, analyzed, and discussed the data. Rob Davidson: Co‐conceived the research plan, reviewed the manuscript, and provided critical feedback on the research plan. Mark Naunton: Edited the manuscript and provided critical feedback on the project and its execution. Chandra Makanjee: Co‐developed and applied the theoretical framework, co‐conceived the research plan, and provided critical insights on the data interpretation and research implications thereof. All authors have read and approved the final version of the manuscript.

## CONFLICT OF INTEREST STATEMENT

The authors have no relevant conflicts of interest to disclose.

## Supporting information



Supporting Information

Supporting Information

Supporting Information

## Data Availability

Authors will share data upon request to the corresponding author.
