# Supplementary material for: Monte Carlo analysis of energy deposition and X‐ray fluence in cylindrical anode systems
Source: J Appl Clin Med Phys. 2025 Sep 30;26(10):e70262. doi: 10.1002/acm2.70262 (PMC12483768; doi:10.1002/acm2.70262)
Supplement: Supplementary file 1 — Supporting Information [file ACM2-26-e70262-s001.docx]

Table 1  **Simulated X-ray fluence, energy deposition, and efficiency**
Results for CNT and Filament beams at 120 keV, showing how polar angle (θₛ) and anode radius (r) affect fluence at the detector, energy deposition on the anode, and conversion efficiency. Values are per incident electron with ±1σ errors.

| Group | Beam Type | Variable | Value | X-ray Fluence at detector (p/cm²/pr) | X-ray Fluence Error (%) | Energy Deposition on anode (GeV/pr) | Energy Deposition Error (%) | Efficiency (%) |
| --- | --- | --- | --- | --- | --- | --- | --- | --- |
| Polar Angle Variation | CNT | θₛ | 71.8° | 1.865E-06 | 0.519 | 2.079E-08 | 0.003 | 0.168 |
| Polar Angle Variation | CNT | θₛ | 58.2° | 1.875E-06 | 0.541 | 2.600E-08 | 0.003 | 0.169 |
| Polar Angle Variation | CNT | θₛ | 48.6° | 1.920E-06 | 0.571 | 3.127E-08 | 0.003 | 0.173 |
| Polar Angle Variation | CNT | θₛ | 40.5° | 1.819E-06 | 0.564 | 3.755E-08 | 0.003 | 0.164 |
| Polar Angle Variation | CNT | θₛ | 33.4° | 1.653E-06 | 0.549 | 4.567E-08 | 0.003 | 0.149 |
| Polar Angle Variation | CNT | θₛ | 26.7° | 1.504E-06 | 0.614 | 5.700E-08 | 0.003 | 0.136 |
| Polar Angle Variation | CNT | θₛ | 20.5° | 1.390E-06 | 0.660 | 7.442E-08 | 0.004 | 0.125 |
| Polar Angle Variation | Filament | θₛ | 71.8° | 1.612E-06 | 0.756 | 1.852E-08 | 0.004 | 0.145 |
| Polar Angle Variation | Filament | θₛ | 58.2° | 1.881E-06 | 0.651 | 2.598E-08 | 0.004 | 0.170 |
| Polar Angle Variation | Filament | θₛ | 48.6° | 1.902E-06 | 0.573 | 3.131E-08 | 0.004 | 0.172 |
| Polar Angle Variation | Filament | θₛ | 40.5° | 1.822E-06 | 0.594 | 3.763E-08 | 0.004 | 0.164 |
| Polar Angle Variation | Filament | θₛ | 33.4° | 1.643E-06 | 0.592 | 4.580E-08 | 0.004 | 0.148 |
| Polar Angle Variation | Filament | θₛ | 26.7° | 1.496E-06 | 0.693 | 5.725E-08 | 0.003 | 0.135 |
| Polar Angle Variation | Filament | θₛ | 20.5° | 1.367E-06 | 0.657 | 7.497E-08 | 0.004 | 0.123 |
| Anode Radius Variation | CNT | r | 1.0 cm | 1.864E-06 | 0.517 | 5.005E-08 | 0.003 | 0.168 |
| Anode Radius Variation | CNT | r | 2.0 cm | 1.920E-06 | 0.571 | 1.251E-08 | 0.003 | 0.173 |
| Anode Radius Variation | CNT | r | 3.0 cm | 1.968E-06 | 0.552 | 5.560E-09 | 0.003 | 0.178 |
| Anode Radius Variation | CNT | r | 4.0 cm | 1.973E-06 | 0.509 | 3.127E-09 | 0.003 | 0.178 |
| Anode Radius Variation | CNT | r | 5.0 cm | 2.013E-06 | 0.505 | 2.001E-09 | 0.003 | 0.182 |
| Anode Radius Variation | Filament | r | 1.0 cm | 1.829E-06 | 0.535 | 5.022E-08 | 0.003 | 0.165 |
| Anode Radius Variation | Filament | r | 2.0 cm | 1.905E-06 | 0.539 | 1.252E-08 | 0.003 | 0.172 |
| Anode Radius Variation | Filament | r | 3.0 cm | 1.956E-06 | 0.525 | 5.563E-08 | 0.003 | 0.176 |
| Anode Radius Variation | Filament | r | 4.0 cm | 1.976E-06 | 0.552 | 3.128E-08 | 0.003 | 0.178 |
| Anode Radius Variation | Filament | r | 5.0 cm | 2.019E-06 | 0.567 | 2.002E-09 | 0.003 | 0.182 |
| Flat Anode | CNT | — | — | 1.277E-06 | 0.620 | 3.521E-08 | 0.003 | 0.115 |
| Flat Anode | Filament | — | — | 1.280E-06 | 0.750 | 3.525E-08 | 0.003 | 0.116 |
