# Supplementary material for: Monte Carlo analysis of energy deposition and X‐ray fluence in cylindrical anode systems
Source: J Appl Clin Med Phys. 2025 Sep 30;26(10):e70262. doi: 10.1002/acm2.70262 (PMC12483768; doi:10.1002/acm2.70262)
Supplement: Supplementary file 2 — Supporting Information [file ACM2-26-e70262-s002.docx]

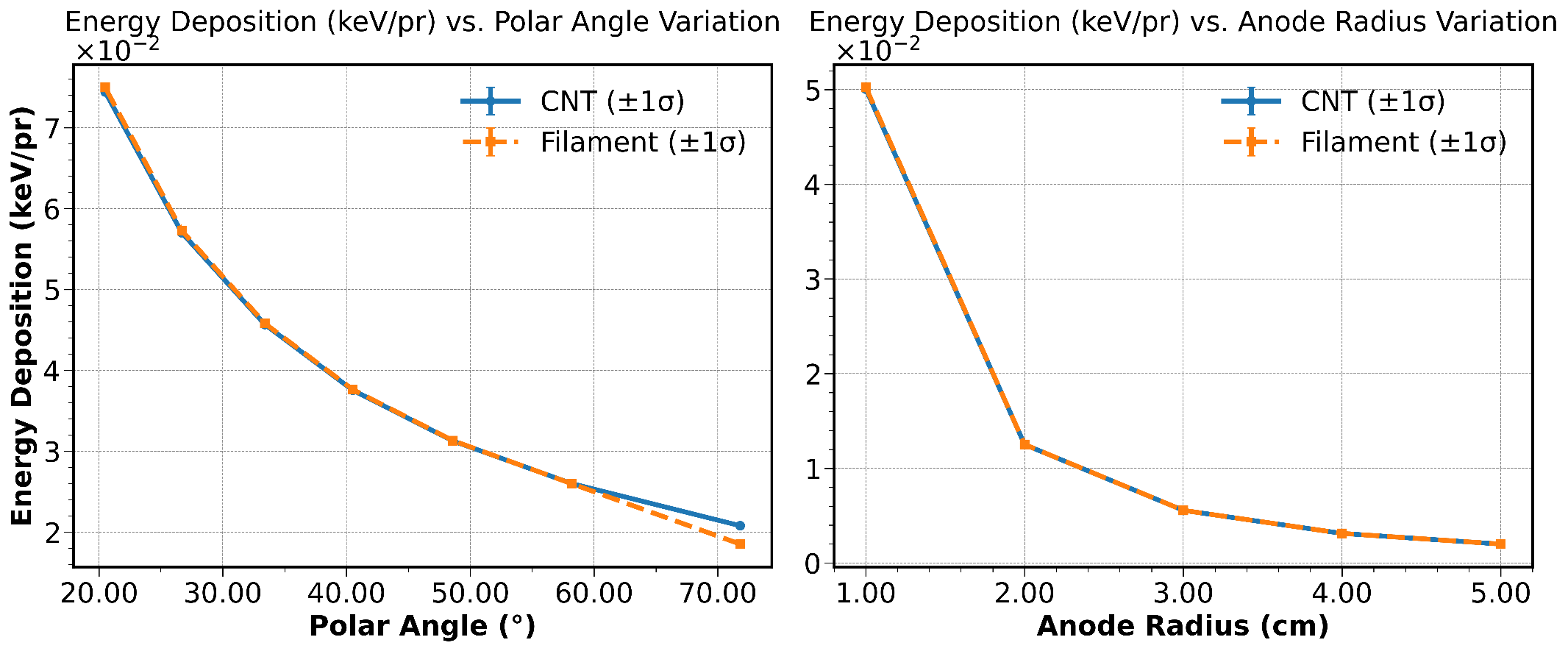


Figure 1 Plots the total energy deposited in the anode, normalized per single incident electron, in units of keV/primary. The left panel shows variation with **polar angle** (°); the right panel shows variation with **anode radius** (cm).
