# Supplementary material for: Monte Carlo analysis of energy deposition and X‐ray fluence in cylindrical anode systems
Source: J Appl Clin Med Phys. 2025 Sep 30;26(10):e70262. doi: 10.1002/acm2.70262 (PMC12483768; doi:10.1002/acm2.70262)
Supplement: Supplementary file 3 — Supporting Information [file ACM2-26-e70262-s003.docx]

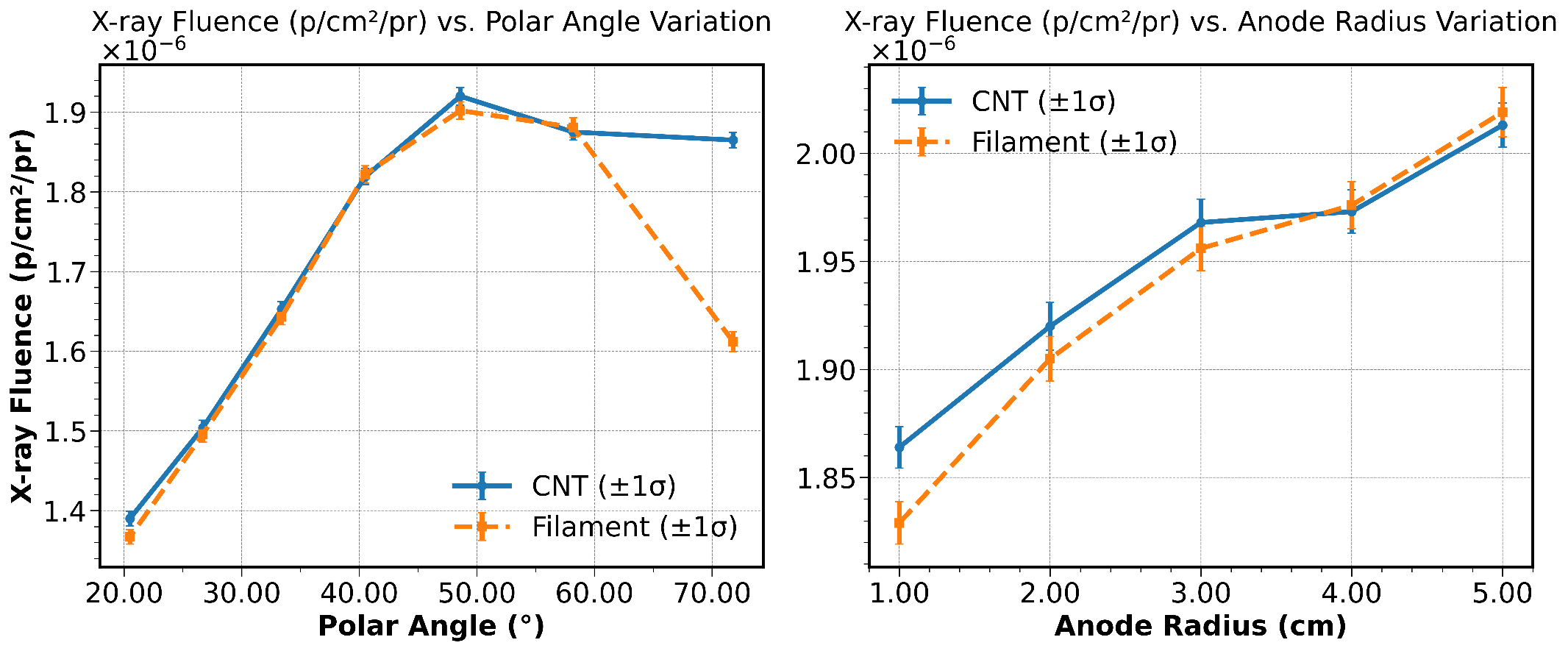


Figure 2 Plots the total photon fluence at the detector plane for photons with energies ≥33 keV, normalized per single incident electron, in units of photons/cm²/primary. The left panel shows variation with polar angle (°) of the beam–anode geometry; the right panel shows variation with anode radius (cm)
